# HTLV-1 tax hijacks cellular ubiquitination machinery to assemble K63-linked polyubiquitin for canonical NF-κB activation

**DOI:** 10.1186/1742-4690-12-S1-O34

**Published:** 2015-08-28

**Authors:** Yik-Khuan A Ho, Huijun Zhi, Tara Bowlin, Batsukh Dorjbal, Subha Philip, Muhammad A Zahoor, Hsiu-Ming Shih, Brian Schaefer, JN Mark Glover, Chou-Zen Giam

**Affiliations:** 1 Department of Microbiology and Immunology, Uniformed Services University of the Health Sciences, Bethesda, MD 20814 USA; 2 Institute of Biomedical Sciences, Academia Sinica, Taipei 115, Taiwan; 3 Department of Biochemistry, Faculty of Medicine and Dentistry, University of Alberta, Edmonton, AB, Canada T6G 2H7

## 

Human T lymphotropic virus type 1 (HTLV-1) trans-activator/oncoprotein, Tax, impacts a multitude of basic cellular processes, including I-κB kinase (IKK) signaling, DNA damage repair, and mitosis. These activities of Tax have been implicated in leukemogenesis, but the underlying mechanisms remain unknown. IKK and its upstream kinase, transforming growth factor β activated kinase-1 (TAK1), contain ubiquitin-binding subunits, NF-κB essential modulator (NEMO) and TAK1 binding protein 2 (TAB2) respectively, which interact with K63-linked polyubiquitin. On this signaling platform, auto-phosphorylation and activation of TAK1 occurs, followed by TAK1-catalyzed IKK phosphorylation and activation. Here we demonstrate in vitro and in vivo that Tax stimulates ubiquitin E2 conjugating enzyme Ubc13:Uev1A (or Ubc13:Uev2) and ubiquitin E3 ligase ring finger protein 8 (RNF8) to assemble long and unanchored K63-linked polyubiquitin for TAK1 and IKK activation. The TAK1 so activated by Tax also promotes JNK phosphorylation. The inappropriate activation of RNF8 — an E3 ligase involved in DNA damage repair, cytokinesis, and centrosome function — by Tax can explain the pleiotropic effects of Tax on signaling pathways.

